# Care management for Type 2 diabetes in the United States: a systematic review and meta-analysis

**DOI:** 10.1186/1472-6963-12-72

**Published:** 2012-03-22

**Authors:** Jason S Egginton, Jennifer L Ridgeway, Nilay D Shah, Saranya Balasubramaniam, Joann R Emmanuel, Larry J Prokop, Victor M Montori, Mohammad Hassan Murad

**Affiliations:** 1 Division of Preventive Medicine and the Knowledge and Evaluation Research Unit, 200 First Street SW, 55905 Rochester, MN, USA; 2 Division of Health Care Policy and Research, Mayo Clinic, Rochester, MN, USA; 3 Division of Endocrinology, Mayo Clinic, Rochester, MN, USA; 4 Division of Preventive Medicine, Mayo Clinic, Rochester, MN, USA

**Keywords:** Chronic Care: disease management/care management, Endocrinology: diabetes, Evidence-based medicine/critical review of evidence

## Abstract

**Background:**

This systematic review and meta-analysis aims at assessing the composition and performance of care management models evaluated in the last decade and their impact on patient important outcomes.

**Methods:**

A comprehensive literature search of electronic bibliographic databases was performed to identify care management trials in type 2 diabetes. Random effects meta-analysis was used when feasible to pool outcome measures.

**Results:**

Fifty-two studies were eligible. Most commonly reported were surrogate outcomes (such as HbA1c and LDL), followed by process measures (clinic visit or testing frequency). Less frequently reported were quality of life, patient satisfaction, self-care, and healthcare utilization. Most care management modalities were carved out from primary care. Meta-analysis demonstrated a statistically significant but trivial reduction of HbA1c (weighted difference in means -0.21%, 95% confidence interval -0.40 to -0.03, p < .03) and LDL-cholesterol (weighted difference in means -3.38 mg/dL, 95% confidence interval -6.27 to -0.49, p < .02).

**Conclusions:**

Most care management programs for patients with type 2 diabetes are 'carved-out', accomplish limited effects on metabolic outcomes, and have unknown effects on patient important outcomes. Comparative effectiveness research of different models of care management is needed to inform the design of medical homes for patients with chronic conditions.

## Background

Chronic care delivery continues to represent a challenge to the healthcare system. New ways to deliver and manage care for patients with chronic conditions such as type 2 diabetes emerged in the late 90s, notably the Chronic Care Model [1]. In 2003, a revision formalized the need for care management [2]. Since then, many approaches have emerged to implement the Chronic Care Model, including those emphasizing the components of self management support and links to community resources. However, many care management models are offered in a carved-out approach- not integrated with the rest of the patients' care, and their effectiveness despite their popularity remains unknown. Care management programs in chronic diseases such as diabetes, congestive heart failure and asthma can be associated with reduced length of hospitalization and medical costs and increased proportion of patients receiving appropriate medications and tests [3].

We conducted a systematic review and meta-analysis of recent controlled trials to summarize the emerging evidence of comparative effectiveness of care management modalities applied to the care of patients with type 2 diabetes across pertinent outcomes. Our focus on the last decade and on type 2 diabetes stems from the need to account for the evolving role of care management and to reflect advances in disease management that have favored type 2 diabetes.

## Research design and methods

This report adheres to the preferred reporting items for systematic reviews and meta-analyses statement (the PRISMA statement) [4].

### Literature search strategy

A comprehensive search of several databases (from 1/1/2000 through 9/21/2011) was designed and conducted by a reference librarian with input from the study's principal investigator. The databases included Ovid Medline In-Process & Other Non-Indexed Citations, Ovid MEDLINE, Ovid EMBASE, Ovid Current Contents, Ovid Cochrane Central Register of Controlled Trials, Ovid Cochrane Database of Systematic Reviews, and EBSCO Cumulative Index to Nursing and Allied Health Literature. Controlled vocabulary supplemented with keywords was used to define the concept areas, diabetes, care management, and treatment outcomes, as well as to limit to controlled trials. The detailed search strategy is available in Additional file 1: APPENDIX.

### Inclusion criteria and data extraction

Three reviewers applied the following inclusion criteria to the abstracts, with each reviewer evaluating two-thirds of the search selections to ensure dual coverage of each abstract: (1) Did the study test a care management intervention in the US on patients with type 2 diabetes? (2) Was the intervention group compared to a control group, such as patients who received usual care? We included studies in which the control group received usual care, attention control, low intensity control interventions, or other types of control groups.

Abstracts that were endorsed by at least one reviewer were included after the initial screening. Citations were kept to request full articles if they were ambiguous. After the full articles were retrieved, each of the three reviewers was assigned the full texts, with a duplicative process again employed to assure dual coverage of each article. Each full article was then evaluated on the criteria above. Endorsement by two reviewers reaching consensus was necessary for an article to be included in the final data extraction and analysis. The agreement among reviewers was adequate, (median kappa statistic = .82).

The primary aim of this systematic review is to describe the available care models in terms of delivery method and team composition and the outcomes they reported (process measures such as testing and visit rates, self-care, quality of life, patient satisfaction, healthcare utilization/cost, and as a secondary aim, surrogate outcomes such as HbA1c and LDL-cholesterol).

### Data analysis

#### Delivery method

We assigned each study to a primary delivery type "Office" if the intervention involved primarily interaction or chart review in the medical outpatient setting; "Web" if most interaction took place on the computer or internet; "Telephone" if the intervention took place over the phone or a pager system; and "Education" if the patient received educational information in another setting such as a community-based facilitated diabetes group.

#### Leader type

If one of the primary leaders of the intervention was discernibly a physician, we determined the trial was "Physician Led." If the intervention was led by a multidisciplinary team or an individual in another type of position, such as a diabetes nurse educator, it was categorized "Not Physician Led."

#### Study description

Study attributes were collected for each article including a description of the enrolled participants and the study setting; detail about the type, duration, frequency and mode of the intervention, as well as information about the control condition; and outcome measures.

#### Study quality

To assess the methodological quality of the described studies, we noted the how randomization and allocation concealment were conducted, whether there were important study arm imbalances at baseline or significant loss to follow up.

#### Analysis

We planned to conduct meta-analysis if data were available and appropriate for statistical pooling. For the continuous outcomes of hemoglobin A1c and LDL cholesterol, we estimated the difference in means from each study with 95% confidence interval (CI). The difference in means is estimated as the difference between the change in the intervention arm and the change in the control arm so that a negative value indicates more decline the intervention arm (i.e., a negative value implies results favoring the care management program). 95% CI that overlaps a value of zero implies no statistically significant effect and is analogous to a 2-tailed p value > 0.05. We pooled the differences in means across studies using the random effects model because of anticipated heterogeneity of the included studies (in terms of design, populations and interventions) [5]. The random effects model incorporates within-study heterogeneity in the CI and produces more conservative estimates. A priori subgroup analyses were defined based on team composition (physician led vs not), delivery method (web vs phone vs office vs education) and length of follow up (≤ 1 year vs. > 1 year) and used to explain heterogeneity by conducting a test of interaction [6]. Heterogeneity was evaluated using the I^2^statistic with values > 50% consistent with substantial heterogeneity [7]. Meta-analysis was conducted using Comprehensive Meta-Analysis, version 2 software [8]. When meta-analysis was not feasible, (i.e., no reported hemoglobin A1c or LDL values or missing measures of precision such as standard deviations and sample sizes for two time points), we presented description of the individual studies in terms of design, care model characteristics and conclusions.

## Results

**Figure 1:**
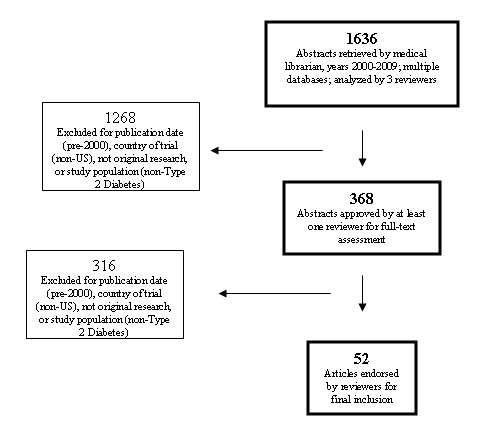
**Systematic review inclusion process**.

Figure 1 displays the results of our search process. The literature search yielded 1636 abstracts from which 52 proved eligible for inclusion.

### Study features

Additional file 1: Table S1 details the characteristics of included studies (median number of enrolled patients is 266, mean study duration is 14 months; and the length of follow-up ranged from a few weeks (49), to 5 years (57). Studies varied by geographic area and clinical team composition.

Criteria for patient enrollment also varied considerably. Some studies focused on specific socioeconomic or demographic characteristics [9-16], while others identified patients based on HbA1c level requirements [12,18-26]. In 11 of the studies, enrolled patients were included or excluded based on HbA1c levels (most typically requiring an elevated HbA1c, e.g., > 8%). A minimum age, or age range, requirement was used in several studies [9,10,13,26-30].

Forty-two of 52 were parallel randomized controlled trials. The other nine studies followed a clustered randomization of physician panels or practices, or were a comparison of two practices. One study was a model-based predictive trial using actual patient data; another looked at before-after data regarding implementation of electronic medical records. The quality of the randomized trials was fair. Some of the methodological limitations noted were lack of reporting of allocation concealment and high or unclear loss to follow up rates. Blinding care givers and patients in this evaluation was not feasible as expected. Cluster randomized trials did not clearly address the issue of contamination. The non-randomized studies were in general of good quality with adequate adjustment for confounders and apparent baseline prognostic balance of the two study arms. Study quality is described in Additional file 1: Table S2.

### Delivery method

Office interventions were the most common, being used in 67% of studies. Telephone and education were next, followed by web interventions, which were only used in seven studies (15%). Thirty-nine percent of studies employed more than one type of intervention (office, telephone, web or education). With the 33 office interventions, nearly half also utilized a telephone or education intervention. Intervention delivery methods and outcomes measured are described in Additional file 1: Figure S1. The care management program was carved out of primary care practice in 36/52 (69%) of the studies.

### Intervention leadership

In 15 of the 52 studies in this review, a physician can be discerned as the leader of the intervention; in the other 36, another professional or group of professionals was identified as facilitating the trial. All physician-led interventions were delivered in the office setting (compared to about half of non-physician-led interventions). Two of the physician-led interventions also had a telephone intervention component, and another two had an education component. The distribution of study leadership and the method of delivery are detailed in Additional file 1: Table S3.

### Study outcomes

**Figure 2:**
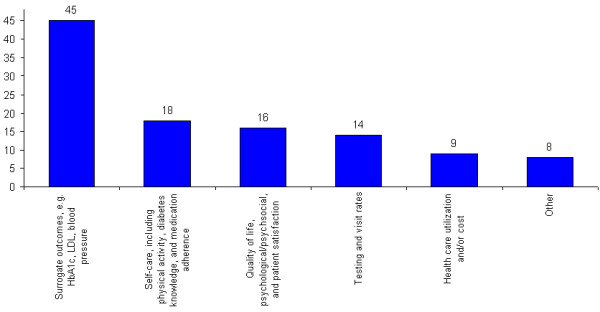
**Types of reported outcomes**.

Figure 2 displays the reported outcomes across studies. Seventy-three percent of studies reported more than one outcome, with an average of two. Additional file 1: Table S4 details the outcomes for each of the 52 studies.

The most commonly reported results were surrogate outcomes, such as HbA1c and LDL-cholesterol. Eighty-five percent of studies included such an outcome, and 30 of those (71%) found statistically significant results. However, in some cases (e.g. Piette et al.) [29], the significance reported was limited to a specific subgroup, the high-risk group. In other cases, the authors only found significant differences between intervention and control groups on a few of the measured surrogate outcomes [13,26,31-36].

Studies also evaluated the impact of CM interventions on process measures (e.g., adherence to disease monitoring protocols such as getting the recommended number of cholesterol tests in eligible populations). Fourteen studies included at least one process outcome, and all of these also had some type of surrogate outcome measurement. Eleven of the 14 studies found some significant results on at least one process measure, but there were mixed results on whether those same studies also achieved desirable surrogate outcomes. In one example, the authors found that a pharmacist-led intervention resulted in significant differences between intervention and control groups on LDL measurement, retinal exam, and foot exam rates [17].

Reductions in HbA1c level were significant, especially among patient with the highest blood levels. In another study, the authors did not find significant results on changes in cholesterol or HbA1c levels, but they did get significant results on 10 American Diabetes Association standards of care measures--a result they surmise may be related to small sample size and short study duration [12]. In another example, [37] improvements in care processes did not translate into improved surrogate outcomes, possibly because baseline levels were relatively good. There were also some associations between outcomes measured.

All but one of the studies that looked at testing or visit rates also measured surrogate outcomes. And in 70 percent of cases, significant results on testing and visit rates were associated with significant surrogate outcomes.

Self-care outcomes were included in nineteen, and ten of these demonstrated positive change. There were a range of self-care measures including foot exams and self-monitoring glucose. Seven of the studies mentioned diet or nutrition as part of the intervention.

There were seventeen studies with quality of life outcomes (including patient satisfaction-related outcomes), with twelve reporting at least one significant improvement. Eight studies included some measure of health care cost or utilization (2 with significant change), although most of them included this as a secondary measure. The "other" category captures outcomes such as the number of risky prescribing events. Only two of those studies achieved statistically significant outcomes.

Physician-led studies primarily focused on surrogate outcomes and measures like visit or testing rates. There were less likely to include outcomes like self-care or quality of life although this comparison is not statistically significant due to the small number of studies (Additional file 1: Figure S2).

### Meta-analysis of HbA1c and LDL outcomes

While 48 studies included some type of surrogate outcome, only 29 provided data sufficient for meta-analysis (seventeen of these reported complete HbA1c measures, twelve reported complete LDL measures, and ten reported both).

**Figure 3:**
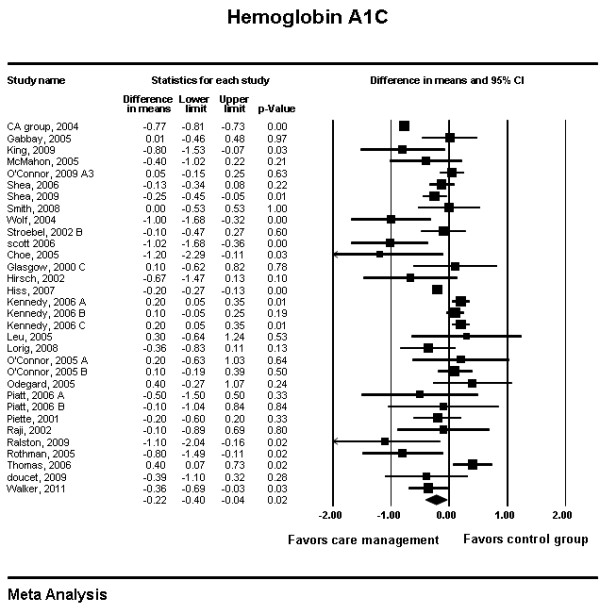
**Meta-analysis of Hemoglobin A1c**.

**Figure 4:**
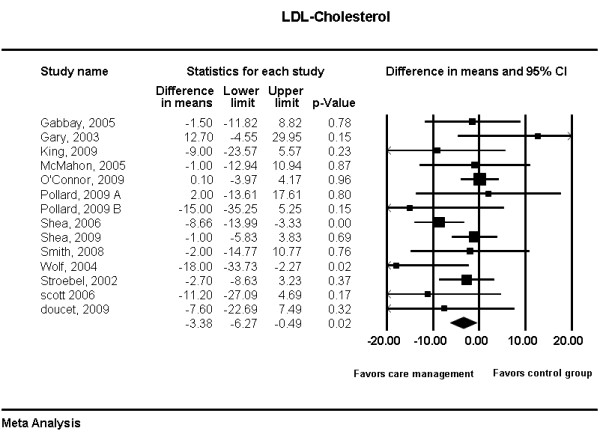
**Meta-analysis of LDL-cholesterol**.

Results of the meta-analyses are shown in Figures 3 and 4. Diabetes care management interventions were associated with a statistically significant reduction in hemoglobin A1c level (weighted difference in means -0.22%; 95% CI -0.40 to -0.04; p = 0.02). This reduction however, was also associated with significant heterogeneity (I^2 ^= 94%) and is unlikely to be clinically important. The trend in reduction in LDL associated with these programs was statistically significant (weighted difference in means - 3.38 mg/dL; 95% CI, -6.27 to -0.49; p = 0.02). This analysis was not associated with significant heterogeneity (I^2 ^= 27%), and was again trivial from a clinical standpoint.

There was no statistically significant interaction for length of intervention and subgroup analysis showed no significant effect for type of intervention (office, web, telephone, or education) or whether a physician played a key role in the intervention. There was no statistically significant interaction for the length of the intervention (defined as > 12 months or ≤ 12 months) for either LDL (p = 0.90) or HbA1c (p = 0.50), (See Additional file 1: Table S5).

## Discussion

We conducted a systematic review of the literature evaluating type 2 diabetes care management interventions in the US during the last decade. We identified 52 studies that demonstrated heterogeneous results in terms of improvements in process measures and paucity of data on patient-important outcomes. We found that these interventions were associated with some improvement in surrogate outcomes (trivial reduction in hemoglobin A1c and LDL cholesterol levels). We could not identify a particular intervention type or team characteristic that is more effective.

In the available literature, many of the disease management programs were carved out of primary care practice. Traditionally, carve out programs were thought to provide the expertise that would be prohibitively expensive for health plans to develop and sustain [38]. However, this trend has changed and health practices are bringing back these programs in-house. Some of the reasons behind this change are the availability of information technology that enables the integration of databases (prescription, claims, and laboratory data); the potential misalignment of vendor and client interests; insufficient transparency and insufficient evidence of improved outcomes [38]. Moreover, integrated care is intuitively more patient-centered and may reduce redundancy. The data synthesized in this report are insufficient to recommend for or against either approach (carved vs not). Overall, the wide variations in intervention delivery methods, duration, and populations, and the use of surrogate outcomes in the included studies, further challenge inference and does not provide evidence to support the use of these models in type 2 diabetes.

The Chronic Care Model identifies the essential elements of a health care system that encourage high-quality chronic disease care. These elements are the community, the health system, self-management support, delivery system design, decision support and clinical information systems [1,2]. The available evidence is derived from programs that do not offer all or most of these components. Further, the studies summarized in this review do not address the recent themes of contemporary chronic care model (patient safety, cultural competency, care coordination, community policies and case management) [1,2]. Therefore; the efficacy of a true and practical chronic care model in type 2 diabetes remains undetermined.

### Comparison with other reviews

Pimouguet et al. conducted a systematic review and meta-analysis focusing on HbA1c and found that care management programs were associated with a statistically significant reduction of -0.38 (-0.47 to -0.29) [39]. This effect size is similar to the one detected in the current analysis although in their analysis they pooled type 1 and type 2 diabetes studies. They found similar rates of hypoglycemia although this outcome was poorly and non systematically reported. Several other older reviews (1999-2006) also focused on A1c but some found improvements in the rates of screening for diabetic retinopathy, foot lesions, peripheral neuropathy, and proteinuria; and on the monitoring of lipid concentrations [40-42]. The current systematic review updates the evidence base and adds to the emerging literature on the impact, or lack thereof, of care management on patient-important outcomes.

A recent randomized trial by McCall et al. [43] assigned 242,417 patients with heart failure or diabetes to eight commercial disease-management programs using nurse-based call centers or to usual care (control). They demonstrated that the intervention did not reduce hospital admissions or emergency room visits but led to 14 (out of 40) significant improvements in process-of-care measures. These modest improvements came at substantial cost to the Medicare program ($400 million) with no demonstrable savings in expenditures. This trial underscores the apparent limits to the premise of care/disease management for diabetes, particularly when provided carved out of the context of primary care.

### Limitations and strengths

The strength of this review stems from the focused clinical question and comprehensive literature search. We were more interested in the recent trend over the past decade in diabetes care management programs and therefore did not evaluate prior publications. We attempted to synthesize the evidence on outcomes that matter to patients although meta-analyses were only possible on surrogate laboratory outcomes. The likelihood of reporting bias and publication bias threatens the validity of this review and its presence is suggested by the selective reporting of outcomes (only a small numbers of studies reported each outcome). This review focuses on the last decade because we aimed at providing a contemporary evaluation of the current trends in these care models. Therefore, our conclusions may be biased since relevant older publications, summarized elsewhere, are not presented in this report. The evaluation of publication bias was not statistically feasible due to the small number of studies and the significant heterogeneity in results across trials [44]. Lastly, we used a consensus process to categorize study intervention methods to allow comparisons across these methods. However, assigning intervention methods to mutually exclusive categories could have biased the observed effects toward the null.

## Conclusion

Best available evidence offers limited certainty about the impact of care management for patients with type 2 diabetes. Based on this limited evidence, care management improves process measures and also improves surrogate outcomes to a trivial extent. Despite that some of the included trials were of sufficient size and duration, there is almost no data regarding benefits of care management on patient-important outcomes, such as living longer and independently, feeling better, or suffering fewer complications.

In conclusion, the current literature does not allow the confident endorsement of a single model or delivery method for care management for patients with type 2 diabetes. Further research is needed to evaluate whether existing models achieve more than the ever improving usual care to improve outcomes of importance to patients.

## Competing interests

The authors declare that they have no competing interests.

## Authors' contributions

MHM and JSE had full access to all of the data in the study and take responsibility for the integrity of the data and the accuracy of the data analysis. MHM, VMM and NDS conceived and designed the study. SB, JRE and LJP acquired the data. MHM and JSE analyzed and interpreted the data. MHM and JSE drafted the manuscript. All authors critically revised the manuscript for important intellectual content. MHM, JSE and JLR provided administrative, technical, or material support. MHM supervised the study. All authors have given final approval for publication.

## Pre-publication history

The pre-publication history for this paper can be accessed here:

http://www.biomedcentral.com/1472-6963/12/72/prepub

## Supplementary Material

Additional file 1 **Table S1**. Study Description, N = 52 [45-66]. Click here for file
