# Supplementary material for: Care management for Type 2 diabetes in the United States: a systematic review and meta-analysis
Source: BMC Health Serv Res. 2012 Mar 22;12:72. doi: 10.1186/1472-6963-12-72 (PMC3349574; doi:10.1186/1472-6963-12-72)
Supplement: Additional file 1 — Table S1. Study Description, N = 52 [45-66]. [file 1472-6963-12-72-S1.DOC]

**Appendix**

*Supplemental table 1—Study Description, N=52*

| **Reference** | **Patients, No.1** | **Population** | **Setting** | **Description** | **Study Duration (mo.)** |
| --- | --- | --- | --- | --- | --- |
| Amoako et al. | 68 | African-American women age 49 plus | Physicians' offices in Guildord County, NC | Psychoeducational telephone intervention to manage diabetes self-care-related uncertainty | 1.5 |
| Batik et al. | 135 | Age 65 plus from low-income, ethnic minority neighborhood | Community clinics, senior center and social services provider, and university research center | Telephone-based motivational support program for physical activity, delivered by older adult volunteers | 6 |
| The California Medi-Cal Type 2 Diabetes Study Group | 362 | Low-income ethnic minority | Clinical sites in southern CA | Intensive diabetes case management interventions | 25 |
| Choe et al. | 120 | HbA1c levels = 8% | University-affiliated ambulatory care clinic in MI | Pharmacist-guided clinic visits and telephone follow-up | 24 |
| Clancy et al. | 120 | Largely African-American, female, and underinsured with HbA1c levels = 8.5% | Adult Primacy Care Center at the Medical University of South Carolina | Group visits with primary care internal medicine physician and diabetes nurse educator | 6 |
| Doucette et al. | 78 | Adult type 2 diabetics | North Carolina Community pharmacy | Education sessions followed by pharmacist counseling on medications, goals and self-care activities | 12 |
| Elder et al. | 60 | New registrants to diabetes registry who had HbA1c between 6.0 and 8.0% | Group model health maintenance organization | Ayurvedic intervention for newly-diagnosed patients: herbal supplement, meditation instruction, diet, and exercise | 6 |
| Gabbay et al. | 332 | Patients with two or more visits for diabetes in past year | Primary care clinics in Penn State Hershey Medical Center | Nurse case management, including self-management education, and implementation of diabetes guidelines | 12 |
| Gary et al. | 186 | Urban African-Americans, age 35 to 75 | Johns Hopkins Outpatient Center or East Baltimore Medical Center | Nurse case manager (NCM), community health worker, or NCM/CHW team | 24 |
| Gary et al. | 542 | Urban African-Americans age 25 plus | University managed care organization, some sites in medically underserved areas | Minimal telephone with trained lay health educator or intensive intervention implemented by a NCM/CHW team | 6 |
| Glasgow and Toobert | 320 | Age 40 plus | The Center for Healthy Living and participating offices | Brief behavioral dietary intervention, telephone follow-up calls and community resources enhancement | 6 |
| Glasgow et al. | 886 | Age 25 plus | Family physicians and general internists insured by Copic Insurance Company in CO | Computer-based intervention at physician's office with consultation by nurse case manager, and follow-up phone call by care manager | 12 |
| Hirsch et al. | 109 | At least two visits with HbA1c levels | Academic family practice clinics | Pharmacist case management , nurse and nutritionist counseling, provider didactics and computerized compliance feedback | 14 |
| Hiss et al. | 197 | Age 18 plus in the southeast MI area that surrounds the city of Detroit | Community-based primary care physicians not affiliated with a managed care organization (“clinically isolated”) | Intervention included baseline meeting with nurse to set short term goals, subsequent meetings to set long term goals, and discussion between nurse and physician | 6 |
| Ilag et al. | 174 | Age 18 plus, enrolled in the managed care organization for at least one year | University affiliated primary care internal medicine practices affiliated with a managed care organization in MI | American Diabetes Assessment Program assessments were completed, reviewed with subjects, mailed to providers, and incorporated into electronic medical record | 24 |
| Izquierdo et al. | 338 | Medicare beneficiaries age 55 plus who reside in federally-designated underserved areas | Telemedicine case management intervention | Detection and response to medically urgent situations using telemedicine case management | 36 |
| Kennedy et al. | 7,893 | Age 19 plus with type 2 diabetes uncontrolled by oral antidiabetic agents and requiring insulin | Nationwide sample from 2,164 clinical sites in the US | Active versus usual titration and point of care versus lab measurement | 6 |
| King and Wolfe | 135 | Age 18 plus with type 2 diabetes and one or more cardiovascular risk factor | Primary care practices within 100 miles of Salinas, CA | Standardized algorithms and education disseminated through computer-assisted and traditional distance medicine methods | 12 |
| Krein et al. | 246 | HbA1c levels > 7.5% | VA hospital in Michigan | Active nurse case management through telephone contact, algorithms, and goal setting | 19 |
| Leu et al. | 50 | HbA1c levels between 8.0 and 9.4% | Neighborhood clinics from the University of Washington Physician's Network | Wireless two-way pager-based automated message system | 5 |
| Lorig et al. | 567 | Spanish-speaking adults age 18 plus | Community settings in six San Francisco Bay Area counties | Six-week community-based, peer-led Spanish Diabetes Self-Management Program with either 15 months of automated telephone messages or no reinforcement | 1.5 |
| McMahon et al. | 104 | Age 18 plus with HbA1c levels = 9.0% | Department of Veterans Affairs Boston Healthcare System | Diabetes education class followed by web-based care management, including messaging with care manager | 12 |
| Nuovo | 395 | Ages 35 to 77 who had not had specific appointment for their DM in the past year | UC Davis Health Systems Primary Care Clinic in Rancho Cordova, CA | Provided results and recommendations, met with physician for 15-minute focused appointment and then with a diabetic nurse educator | 6 |
| O’Connor et al. | 122 | Age 18 plus | Primary care clinics in the HealthPartners Medical Group, Minnesota | Electronic medical record provided basic decision support, including prompts and reminders for diabetes care | 60 |
| O’Connor et al. | 754 | Practices with no current commitment to other organized improvement efforts and patients age 19 plus | Primary care medical practices with medical group participation in HealthPartners insurance products | Provider intervention using IDEAL (Improving Care for Diabetes Through Empowerment, Active Collaboration, and Leadership) model and seven-step quality improvement process | 18 |
| O’Connor et al. | 3,703 | All patients in study clinics who met study criteria for diagnosis of diabetes | Primary care practices in HealthPartners Medical Group, Minnesota | Intervention arms included customized feedback of clinical information for patient, physician, or both (neither for control group) | 36 |
| O’Connor et al. | 2,020 | Age 19 plus in study clinics who met study criteria for diagnosis of diabetes | Primary care practices in HealthPartners Medical Group, Minnesota | Physician intervention with simulated case-based learning intervention, or same intervention plus physician opinion leader feedback | 12 |
| Odegard et al. | 77 | Age 18 plus with HbA1c levels = 9% and taking at least one oral diabetes medication | University of Washington Medicine Clinics (pharmacists not working in clinics) | Development of a diabetes care plan, regular pharmacist-patient communication on diabetes care progress, and pharmacist-provider communication on the subject’s care progress | 12 |
| Patric et al. | 2,160 | Age 18 plus | TennCare managed care organizations | Diabetes case management including quarterly educational mailings to participants, quarterly provider office visits, provider report card mailings, and patient reminders | 12 |
| Pettitt et al. | 240 | Age 18 plus in counties with low-income ethnic minority populations | Clinical sites in Santa Barbara, Los Angeles, and San Diego counties in CA | Intensive diabetes case management, contacting patients at varying intervals according to need (at least monthly) to lower A1c | 23 |
| Phillips et al. | 4,138 | Economically disadvantaged municipal population | Grady Medical Clinic, the largest site of primary care in the Grady Health System | Hard copy computerized reminders with patient-specific recommendations at the time of visit, individual face-to-face feedback on performance for 5 minutes every 2 weeks, or both | 36 |
| Piatt et al. | 119 | Underserved urban suburb of Pittsburg, PA | Primary care practices | A community care model (CCM) intervention with patient and provider education, as well as other CCM elements in the community, or a provider education-only intervention | 6 |
| Piette et al. | 272 | Patients with diabetes using hypoglycemic medications | General medicine clinics and diabetes specialty clinic in Department of Veterans Affairs health care system | Automated telephone disease management with telephone nurse follow-up | 12 |
| Pitale et al. | 153 | Male veterans age 40-69 | Five Department of Veterans Affairs medical centers | Intensive glucose control and monthly visits versus standard treatment and quarterly visits | 24 |
| Pollard et al. | 661 | Patients with two years continuous care | Six Federally Qualified Health Centers in rural WV | Medical centers grouped by low, medium or high use of patient registry | 24 |
| Quinn et al. | 260 | Age 18 to 64, with HbA1c levels = 7.5% | Physician practices in four geographic areas of MD, including urban, suburban, and rural areas | Diabetes communication system, using mobile phones and patient-physician portals to allow patient-specific treatment and communication | 12 |
| Raji et al. | 106 | Age 18 plus with HbA1c levels = 8.5% | Veterans Affairs Boston Healthcare System | Intensive group education (lectures, group discussions, and supervised exercise) versus passive education (mailed educational material) | 12 |
| Ralston et al. | 83 | Age 18 to 75 with HbA1c levels = 7% | University of Washington General Internal Medicine Clinic | Web-based care management , including patient access to electronic medical records, secure email with providers, feedback on blood readings, educational website, and online diary | 12 |
| Rothman et al. | 217 | Age 18 plus with HbA1c levels = 8% | University of North Carolina General Internal Medicine Practice | Pharmacist-led disease management program with intensive education sessions, evidence-based algorithms, and proactive management | 12 |
| Samuel-Hodge et al. | 205 | African American adults with type 2 diabetes | Twenty-four Churches in North Carolina | Counseling and group sessions, phone and postcard contacts | 8 |
| Scott et al. | 149 | Adults with type 2 diabetes | Community Health Center in Iowa | Appointments with a pharmacist at baseline and at 3, 6, and 9 months. | 9 |
| Schmittdiel et al. | 179,249 | Age 20 to 85 and meeting case management criteria | Kaiser Permanente Northern California integrated healthcare delivery system | Diabetes care management, including intensive counseling on medication management, and adherence, diet and lifestyle | 12 |
| Sequist et al. | 6,243 | Patients with diabetes or coronary artery disease and at least one overdue health service | Network of outpatient clinics, community hospitals, and two academic teaching hospitals, in the Boston, MA, area | Integrated, patient-specific electronic clinical reminder system, with reminders automatically displayed each time the clinician opened the patient chart | 6 |
| Shea et al. | 1,665 | Medicare beneficiaries age 55 plus, residing in a federally designated medically underserved area | Primary care practices in New York City and upstate New York | Home telemedicine unit with videoconferencing, remote monitoring, and access to web portal and educational website | 12 |
| Shea et al. | 1,665 | Medicare beneficiaries age 55 plus, residing in a federally designated medically underserved area | Primary care practices in New York City and upstate New York | Home telemedicine unit with videoconferencing, remote monitoring, and access to web portal and educational website | 60 |
| Smith et al. | 639 | Residents of Olmsted County, MN | Six primary care practices affiliated with Mayo Clinic’s site in Rochester, MN | At first patient referral, physician received a telemedicine intervention. After subsequent clinical encounters an endocrinologist reviewed an abstract from the electronic medical record and provided management recommendations. | 30 |
| Stroebel et al. | 1,083 | Participating physicians’ patient panels (Olmsted County, MN) | The Division of Community Internal Medicine at Mayo Clinic in Rochester, MN | Interventions for three groups of physicians including combination of academic detailing, hot lists, team time, and automatic letters | 6 |
| Thomas and Miceli | 347 | Age 18 plus with type 2 diabetes, hypertension, or both, and not at goal | Large employer group at two Lockheed Martin sites (GA and MS) | *Know Your Health* patient education program, as well as encouragement to join on-site fitness facility | 6 |
| Walker et al. | 526 | Urban minorities with HbA1c levels > 7.5% | Members of a health benefit plan in NY/NJ, mean age ~56 | Randomized trial comparing telephonic intervention by health educators versus print | 12 |
| Whitlock et al. | 28 | HbA1c levels > 8% | Internal medicine, primary care, family practice clinics at Eisenhower Army Medical Center | Telemedicine intervention with nurse case manager contact once a week and primary care physician contact once a month | 3 |
| Williams et al. | 866 | Type 2 diabetes patients age 25 plus | Kaiser Permanente Colorado and Copic Insurance | Computer touch screen assessment and individualized self-management action plan | 12 |
| Wolf et al. | 147 | Patients in VA age 20 plus with diabetes and obesity and using diabetes medications | University of Virginia Health System and General Clinical Research Center | Registered dietician met with participants individually, in groups, and by phone for assessment, goal setting, education and support | 12 |

1= Intervention group(s) *n* + control group(s) *n*

Supplemental table 2—*Quality of Studies, N=52*

2A. Randomized

| **Reference** | **Study Design** | **Quality** |
| --- | --- | --- |
| Amoako et al. | Randomized, controlled | Baseline differences in age and diabetes more than two years. Outcome measured only two weeks after intervention. |
| Batik et al. | Cluster Randomized | Low numbers offered intervention during clinic visits. Intervention and control ages significantly different. |
| The California Medi-Cal Type 2 Diabetes Study Group | Randomized, controlled | Arms balanced at baseline. Unexpected improvement in control group. |
| Choe et al. | Randomized, controlled, nonblinded | Arms balanced at baseline. Substantial differential dropout. |
| Clancy et al. | Randomized, controlled, clinical personnel blinded assignment (unless told by patient) | Arms balanced at baseline |
| Doucette | Randomized, controlled | Arms balanced at baseline |
| Elder et al. | Randomized, technicians blinded | Low mean HbA1c at baseline out of necessity. Unable to blind participants. |
| Gabbay et al. | Randomized, controlled | Arms balanced at baseline |
| Gary et al. | Cluster Randomized | Baseline HbA1c lower for participants than nonresponders. Baseline characteristics similar across trial groups. Small number of participants recruited and low participation rates. |
| Gary et al. | Randomized | Arms balanced at baseline |
| Glasgow and Toobert | Randomized, controlled | Arms balanced at baseline |
| Glasgow et al. | Stratified randomized, controlled | Arms balanced at baseline. Lack of electronic medical records data. |
| Hiss et al. | Randomized, controlled | Arms balanced at baseline |
| Ilag et al. | Randomized, controlled, nonblinded | Arms balanced at baseline. University-affiliated IM practices with good compliance with clinical practice guidelines at baseline. |
| Izquierdo et al. | Randomized | No reliable mechanism to identify medically urgent situations in control group |
| Kennedy et al. | Randomized | Arms balanced at baseline. Large nationwide patient sample. |
| Krein et al. | Randomized, controlled | Control group rates slightly poorer health at baseline |
| Leu et al. | Randomized, controlled, nonblinded | Arms balanced at baseline |
| Lorig et al. | Randomized trial and randomized comparison of reinforcement versus no reinforcement. Nonblinded. | Moderately high noncompletion rate but few differences at baseline. No control group for reinforcement trial. |
| McMahon et al. | Random assignment after initial education session | Open-label design. Low prevalence of women recruited to study. |
| O’Connor et al. | Randomized, controlled | Able to recruit less than 15% of primary care clinics within sampling frame. Control group agreed to abstain from competing initiatives during study period. |
| O’Connor et al. | Random assignment of clinics in a single medical group. Controlled. | Some baseline differences by clinic (covariate adjustment in subsequent analyses). Lack of electronic medical records data. |
| O’Connor et al. | Random assignment of physicians (blocked into groups) in a single medical group. Pre-test/post-test control group design. | Similar patient samples except that those in group B more often had coronary artery disease and higher Charlson scores. Conducted at a site with relatively good baseline diabetes care. |
| Odegard et al. | Randomized, controlled, multi-clinic | Baseline differences in high school education and difficulty in remembering to take medications. Randomized within clinics, so providers may have cared for patients in both groups. |
| Pettitt et al. | Randomized, controlled | Arms balanced at baseline except for follow-up time |
| Phillips et al. | Randomized, controlled assignment of residents | Intervention arms balanced at baseline. Adjusted for control differences in follow-up and Medical Clinic visits. Possibility of contamination. |
| Piatt et al. | Multi-level, nonblinded, cluster-design, randomized, controlled | Arms balanced at baseline except for age. Adjusted for usual care group having lower baseline HbA1c levels. |
| Piette et al. | Randomized, controlled | Mostly similar characteristics at baseline but intervention patients more likely to be white, have somewhat more complications, and be seen in ophthalmology clinics in past year. Follow-up by various nurses working under somewhat different constraints. |
| Pitale et al. | Randomized, prospective trial | Arms balanced at baseline |
| Quinn et al. | Random assignment of physician practices | Not described |
| Raji et al. | Cluster randomized | Not described |
| Ralston et al. | Open, randomized, single-center, controlled trial with a parallel group design. Nonblinded after randomization. | Arms balanced at baseline |
| Rothman et al. | Randomized, controlled | Mostly similar characteristics at baseline but intervention patients were slightly older and more likely to be African American. Conducted at a single academic medical center. Follow-up imperfect. |
| Samuel-Hodge | Randomized, controlled | The minimal intervention group was ~4y older (mean, p<.05); otherwise balanced at baseline. |
| Scott | Randomized, controlled | Arms balanced at baseline |
| Sequist et al. | Stratified cluster randomization of clinics. Controlled. | Patients in the intervention and control arms were significantly different in distribution of race and insurance status |
| Shea et al. | Randomized within clusters defined by primary care provider patient panels. Some personnel blinded. Controlled. | Intervention and usual care groups were balanced at baseline, but there were differences by region of recruitment. Differential loss to follow-up. Many physicians managed patients in both the intervention and control groups. |
| Shea et al. | Randomized controlled trial with blinded assessment of the outcomes | Arms balanced at baseline |
| Smith et al. | Cluster randomized | Patient arms balanced at baseline. |
| Stroebel et al. | Cluster randomized | Relatively short duration of intervention and high baseline rates. Management decisions left to discretion of the provider. No control group without access to registry. |
| Thomas and Miceli | Randomized, controlled, nonblinded | More males in control group |
| Walker et al. | Randomized | Arms balanced at baseline |
| Whitlock et al. | Randomized and stratified | Arms balanced at baseline |
| Williams et al. | Cluster randomized controlled | Baseline differences in age and number of chronic conditions |
| Wolf et al. | Randomized controlled. Study personnel blinded until assignment. | Arms balanced at baseline |

2B. Nonrandomized

| **Reference** | **Study Design** | **Quality** |
| --- | --- | --- |
| Hirsch et al. | Controlled, pre-test/post-test, allocation at the firm level | Arms balanced at baseline |
| King and Wolfe | One practice designated for intervention and one for control. In experimental group, all eligible patients invited to participate. | Issues identifying to very similar practices. Baseline differences in HbA1c levels. |
| Nuovo | All eligible patients invited to a planned visit, with those not attending used as comparison group | Arms balanced at baseline |
| O’Connor et al. | Longitudinal study of EMR and non-EMR sites | Clinics similar in size, physician stability, suburban location, and patient baseline HbA1c levels. EMR clinic participated in other diabetes-related care improvement activities during the study period. |
| Patric et al. | Evaluation using a quasi-experimental, nonequivalent control group design | Adjusted for difference between intervention and control group gender, ethnicity, and baseline scores |
| Pollard et al. | Data from pre- and post-registry compared among three treatment and control groups. Non-randomly assigned control group. | Health centers separated into three treatment groups based on the extent that they utilized a patient registry. Reviewers (blind to the data) rated use based on their own observations. |
| Schmittdiel et al. | Observational study with propensity score matching of care management patients to control subjects | Care management program did not always follow specified guidelines for enrolling patients and mean program enrollment was longer than goal |

**References:**

Amoako, Emelia, Anne H. Skelly, and Eileen K. Rossen. 2008. Outcomes of an intervention to reduce uncertainty among African American women with diabetes. *Western Journal of Nursing Research* 30 (8):928-42.

Batik, Odette, Elizabeth A. Phelan, Julie A. Walwick, Grace Wang, and James P. LoGerfo. 2008. Translating a community-based motivational support program to increase physical activity among older adults with diabetes at community clinics: a pilot study of Physical Activity for a Lifetime of Success (PALS). *Preventing Chronic Disease* 5 (1):A18.

California Medi-Cal Type 2 Diabetes Study, Group. 2004. Closing the gap: effect of diabetes case management on glycemic control among low-income ethnic minority populations: the California Medi-Cal type 2 diabetes study.[see comment]. *Diabetes Care* 27 (1):95-103.

Choe, Hae Mi, Sonya Mitrovich, Daniel Dubay, Rodney A. Hayward, Sarah L. Krein, and Sandeep Vijan. 2005. Proactive case management of high-risk patients with type 2 diabetes mellitus by a clinical pharmacist: a randomized controlled trial. *American Journal of Managed Care* 11 (4):253-60.

Clancy, Dawn E., Shirley B. Brown, Kathryn M. Magruder, and Peng Huang. 2003. Group visits in medically and economically disadvantaged patients with type 2 diabetes and their relationships to clinical outcomes. *Topics in Health Information Management* 24 (1):8-14.

Doucette, William R., Matthew J. Witry, Karen B. Farris, and Randal P. McDonough. 2009. Community pharmacist-provided extended diabetes care. *Annals of Pharmacotherapy* 43 (5):882-9.

Elder, Charles, Mikel Aickin, Vance Bauer, Joan Cairns, and Nancy Vuckovic. 2006. Randomized trial of a whole-system ayurvedic protocol for type 2 diabetes. *Alternative Therapies in Health & Medicine* 12 (5):24-30.

Gabbay, Robert A., Irina Lendel, Tipufaiz M. Saleem, Gregory Shaeffer, Alan M. Adelman, David T. Mauger, Mary Collins, and Rosemary C. Polomano. 2006. Nurse case management improves blood pressure, emotional distress and diabetes complication screening. *Diabetes Research & Clinical Practice* 71 (1):28-35.

Gary, Tiffany L., Marian Batts-Turner, Lee R. Bone, Hsin-Chieh Yeh, Nae-Yuh Wang, Felicia Hill-Briggs, David M. Levine, Neil R. Powe, Martha N. Hill, Christopher Saudek, Maura McGuire, and Frederick L. Brancati. 2004. A randomized controlled trial of the effects of nurse case manager and community health worker team interventions in urban African-Americans with type 2 diabetes. *Controlled Clinical Trials* 25 (1):53-66.

Gary, Tiffany L., Lee R. Bone, Martha N. Hill, David M. Levine, Maura McGuire, Christopher Saudek, and Frederick L. Brancati. 2003. Randomized controlled trial of the effects of nurse case manager and community health worker interventions on risk factors for diabetes-related complications in urban African Americans. *Preventive Medicine* 37 (1):23-32.

Glasgow, R. E., P. A. Nutting, D. K. King, C. C. Nelson, G. Cutter, B. Gaglio, A. K. Rahm, and H. Whitesides. 2005. Randomized effectiveness trial of a computer-assisted intervention to improve diabetes care. *Diabetes Care* 28 (1):33-39.

Glasgow, R. E., and D. J. Toobert. 2000. Brief, computer-assisted diabetes dietary self-management counseling: effects on behavior, physiologic outcomes, and quality of life.[see comment]. *Medical Care* 38 (11):1062-73.

Hirsch, Irl B., Harold I. Goldberg, Allan Ellsworth, Timothy C. Evans, Christian D. Herter, Scott D. Ramsey, Mary Mullen, William E. Neighbor, and Allen D. Cheadle. 2002. A multifaceted intervention in support of diabetes treatment guidelines: a cont trial. *Diabetes Research & Clinical Practice* 58 (1):27-36.

Hiss, Roland G., Betty A. Armbruster, Mary Lou Gillard, and Leslie A. McClure. 2007. Nurse care manager collaboration with community-based physicians providing diabetes care: a randomized controlled trial. *Diabetes Educator* 33 (3):493-502.

Ilag, Liza L., Catherine L. Martin, Bahman P. Tabaei, Deanna J. M. Isaman, Ray Burke, Douglas A. Greene, and William H. Herman. 2003. Improving diabetes processes of care in managed care.[see comment]. *Diabetes Care* 26 (10):2722-7.

Izquierdo, R., S. Meyer, J. Starren, R. Goland, J. Teresi, S. Shea, and R. S. Weinstock. 2007. Detection and remediation of medically urgent situations using telemedicine case management for older patients with diabetes mellitus. *Therapeutics and Clinical Risk Management* 3 (3):485-489.

Kennedy, L., W. H. Herman, P. Strange, and A. Harris. 2006. Impact of active versus usual algorithmic titration of basal insulin and point-of-care versus laboratory measurement of HbA<sub>1c</sub> on glycemic control in patients with type 2 diabetes: The Glycemic Optimization with Algorithms and Labs at Point of Care (GOAL A1C) trial. *Diabetes Care* 29 (1):1-8.

King, Allen B., and Gary S. Wolfe. 2009. Evaluation of a diabetes specialist-guided primary care diabetes treatment program. *Journal of the American Academy of Nurse Practitioners* 21 (1):24-30.

Krein, Sarah L., Mandi L. Klamerus, Sandeep Vijan, Jan L. Lee, James T. Fitzgerald, Alan Pawlow, Pamela Reeves, and Rodney A. Hayward. 2004. Case management for patients with poorly controlled diabetes: a randomized trial.[see comment]. *American Journal of Medicine* 116 (11):732-9.

Leu, M. G., T. E. Norris, J. Hummel, M. Isaac, and M. W. Brogan. 2005. A randomized, controlled trial of an automated wireless messaging system for diabetes. *Diabetes Technology and Therapeutics* 7 (5):710-718.

Lorig, Kate, Philip L. Ritter, Frank Villa, and John D. Piette. 2008. Spanish diabetes self-management with and without automated telephone reinforcement: two randomized trials. *Diabetes Care* 31 (3):408-14.

McMahon, Graham T., Helen E. Gomes, Sara Hickson Hohne, Tang Ming-Jye Hu, Betty A. Levine, and Paul R. Conlin. 2005. Web-based care management in patients with poorly controlled diabetes.[see comment]. *Diabetes Care* 28 (7):1624-9.

Nuovo, J. 2009. The impact of planned visits on patients with type 2 diabetes mellitus. *Clinical Medicine: Endocrinology and Diabetes* 2009 (2):7-14.

O'Connor, P. J., J. Sperl-Hillen, P. E. Johnson, W. A. Rush, and A. L. Crain. 2009. Customized feedback to patients and providers failed to improve safety or quality of diabetes care: A randomized trial. *Diabetes Care* 32 (7):1158-1163.

O'Connor, Patrick J., A. Lauren Crain, William A. Rush, Joann M. Sperl-Hillen, Jay J. Gutenkauf, and Jane E. Duncan. 2005. Impact of an electronic medical record on diabetes quality of care. *Annals of Family Medicine* 3 (4):300-6.

O'Connor, Patrick J., Jay Desai, Leif I. Solberg, Laurel A. Reger, A. Lauren Crain, Stephen E. Asche, Teresa L. Pearson, Cynthia K. Clark, William A. Rush, Linda M. Cherney, Joann M. Sperl-Hillen, and Donald B. Bishop. 2005. Randomized trial of quality improvement intervention to improve diabetes care in primary care settings. *Diabetes Care* 28 (8):1890-7.

O'Connor, Patrick J., Joann M. Sperl-Hillen, Paul E. Johnson, William A. Rush, Stephen E. Asche, Pradyumina Dutta, and George R. Biltz. 2009. Simulated physician learning intervention to improve safety and quality of diabetes care: a randomized trial. *Diabetes Care* 32 (4):585-90.

Odegard, Peggy S., Alvin Goo, Jeff Hummel, Kristal L. Williams, and Shelly L. Gray. 2005. Caring for poorly controlled diabetes mellitus: a randomized pharmacist intervention. *Annals of Pharmacotherapy* 39 (3):433-40.

Patric, K., J. D. Stickles, R. S. Turpin, J. B. Simmons, J. Jackson, E. Bridges, and M. Shah. 2006. Diabetes disease management in medicaid managed care: A program evaluation. *Disease Management* 9 (3):144-156.

Pettitt, David J., Alison Okada Wollitzer, Lois Jovanovic, Guozhong He, and Eli Ipp. 2005. Decreasing the risk of diabetic retinopathy in a study of case management: the California Medi-Cal Type 2 Diabetes Study. *Diabetes Care* 28 (12):2819-22.

Phillips, Lawrence S., David C. Ziemer, Joyce P. Doyle, Catherine S. Barnes, Paul Kolm, William T. Branch, Jane M. Caudle, Curtiss B. Cook, Virginia G. Dunbar, Imad M. El-Kebbi, Daniel L. Gallina, Risa P. Hayes, Christopher D. Miller, Mary K. Rhee, Dennis M. Thompson, and Clyde Watkins. 2005. An endocrinologist-supported intervention aimed at providers improves diabetes management in a primary care site: improving primary care of African Americans with diabetes (IPCAAD) 7. *Diabetes Care* 28 (10):2352-60.

Piatt, Gretchen A., Trevor J. Orchard, Sharlene Emerson, David Simmons, Thomas J. Songer, Maria M. Brooks, Mary Korytkowski, Linda M. Siminerio, Usman Ahmad, and Janice C. Zgibor. 2006. Translating the chronic care model into the community: results from a randomized controlled trial of a multifaceted diabetes care intervention.[see comment]. *Diabetes Care* 29 (4):811-7.

Piette, J. D., M. Weinberger, F. B. Kraemer, and S. J. McPhee. 2001. Impact of automated calls with nurse follow-up on diabetes treatment outcomes in a Department of Veterans Affairs health care system: A randomized controlled trial. *Diabetes Care* 24 (2):202-208.

Pitale, Shailesh, Diane Kernan-Schroeder, Nicholas Emanuele, Clark Sawin, Jerome Sacks, Carlos Abraira, and Vacsdm Study Group. 2005. Health-related quality of life in the VA Feasibility Study on glycemic control and complications in type 2 diabetes mellitus. *Journal of Diabetes & its Complications* 19 (4):207-11.

Pollard, Cecil, Kelly A. Bailey, Trisha Petitte, Adam Baus, Mary Swim, and Michael Hendryx. 2009. Electronic patient registries improve diabetes care and clinical outcomes in rural community health centers. *Journal of Rural Health* 25 (1):77-84.

Quinn, C. C., A. L. Gruber-Baldini, M. Shardell, K. Weed, S. S. Clough, M. Peeples, M. Terrin, L. Bronich-Hall, E. Barr, and D. Lender. 2009. Mobile diabetes intervention study: Testing a personalized treatment/behavioral communication intervention for blood glucose control. *Contemporary Clinical Trials* 30 (4):334-346.

Raji, Annaswamy, Helen Gomes, Judith O. Beard, Patricia MacDonald, and Paul R. Conlin. 2002. A randomized trial comparing intensive and passive education in patients with diabetes mellitus. *Archives of Internal Medicine* 162 (11):1301-4.

Ralston, James D., Irl B. Hirsch, James Hoath, Mary Mullen, Allen Cheadle, and Harold I. Goldberg. 2009. Web-based collaborative care for type 2 diabetes: a pilot randomized trial.[see comment]. *Diabetes Care* 32 (2):234-9.

Rothman, Russell L., Robb Malone, Betsy Bryant, Ayumi K. Shintani, Britton Crigler, Darren A. Dewalt, Robert S. Dittus, Morris Weinberger, and Michael P. Pignone. 2005. A randomized trial of a primary care-based disease management program to improve cardiovascular risk factors and glycated hemoglobin levels in patients with diabetes.[see comment]. *American Journal of Medicine* 118 (3):276-84.

Samuel-Hodge, C. D., T. C. Keyserling, S. Park, L. F. Johnston, Z. Gizlice, and S. I. Bangdiwala. 2009. A randomized trial of a church-based diabetes self-management program for African Americans with type 2 diabetes. *Diabetes Educ* 35 (3):439-54.

Schmittdiel, J. A., C. S. Uratsu, B. H. Fireman, and J. V. Selby. 2009. The effectiveness of diabetes care management in managed care. *American Journal of Managed Care* 15 (5):295-301.

Scott, David M., Steven T. Boyd, Michelle Stephan, Sam C. Augustine, and Thomas P. Reardon. 2006. Outcomes of pharmacist-managed diabetes care services in a community health center. *American Journal of Health-System Pharmacy* 63 (21):2116-22.

Sequist, T. D., T. K. Gandhi, A. S. Karson, J. M. Fiskio, D. Bugbee, M. Sperling, E. F. Cook, E. J. Orav, D. G. Fairchild, and D. W. Bates. 2005. A randomized trial of electronic clinical reminders to improve quality of care for diabetes and coronary artery disease. *Journal of the American Medical Informatics Association* 12 (4):431-437.

Shea, Steven, Ruth S. Weinstock, Justin Starren, Jeanne Teresi, Walter Palmas, Lesley Field, Philip Morin, Robin Goland, Roberto E. Izquierdo, L. Thomas Wolff, Mohammed Ashraf, Charlyn Hilliman, Stephanie Silver, Suzanne Meyer, Douglas Holmes, Eva Petkova, Linnea Capps, and Rafael A. Lantigua. 2006. A randomized trial comparing telemedicine case management with usual care in older, ethnically diverse, medically underserved patients with diabetes mellitus. *Journal of the American Medical Informatics Association* 13 (1):40-51.

Shea, Steven, Ruth S. Weinstock, Jeanne A. Teresi, Walter Palmas, Justin Starren, James J. Cimino, Albert M. Lai, Lesley Field, Philip C. Morin, Robin Goland, Roberto E. Izquierdo, Susana Ebner, Stephanie Silver, Eva Petkova, Jian Kong, Joseph P. Eimicke, and I. DEATel Consortium. 2009. A randomized trial comparing telemedicine case management with usual care in older, ethnically diverse, medically underserved patients with diabetes mellitus: 5 year results of the IDEATel study. *Journal of the American Medical Informatics Association* 16 (4):446-56.

Smith, Steven A., Nilay D. Shah, Sandra C. Bryant, Teresa J. H. Christianson, Susan S. Bjornsen, Paula D. Giesler, Kathleen Krause, Patricia J. Erwin, Victor M. Montori, and Group Evidens Research. 2008. Chronic care model and shared care in diabetes: randomized trial of an electronic decision support system.[erratum appears in Mayo Clin Proc. 2008 Oct;83(10):1189]. *Mayo Clinic Proceedings* 83 (7):747-57.

Stroebel, Robert J., Sidna M. Scheitel, John S. Fitz, Ruth A. Herman, James M. Naessens, Christopher G. Scott, David A. Zill, and Lisa Muller. 2002. A randomized trial of three diabetes registry implementation strategies in a community internal medicine practice. *Joint Commission Journal on Quality Improvement* 28 (8):441-50.

Thomas, Pamella D., and Robert Miceli. 2006. Evaluation of the "Know Your Health" program for type 2 diabetes mellitus and hypertension in a large employer group. *American Journal of Managed Care* 12 Spec no.:SP33-9.

Walker, E. A., C. Shmukler, R. Ullman, E. Blanco, M. Scollan-Koliopoulus, and H. W. Cohen. 2011. Results of a successful telephonic intervention to improve diabetes control in urban adults: A randomized trial. *Diabetes Care* 34 (1):2-7.

Whitlock, W. L., A. Brown, K. Moore, H. Pavliscsak, A. Dingbaum, D. Lacefield, K. Buker, and S. Xenakis. 2000. Telemedicine improved diabetic management. *Military Medicine* 165 (8):579-84.

Williams, Geoffrey C., Martin Lynch, and Russell E. Glasgow. 2007. Computer-assisted intervention improves patient-centered diabetes care by increasing autonomy support. *Health Psychology* 26 (6):728-34.

Wolf, Anne M., Mark R. Conaway, Jayne Q. Crowther, Kristen Y. Hazen, Jerry L Nadler, Beverly Oneida, Viktor E. Bovbjerg, and Study Improving Control with Activity and Nutrition. 2004. Translating lifestyle intervention to practice in obese patients with type 2 diabetes: Improving Control with Activity and Nutrition (ICAN) study.[see comment]. *Diabetes Care* 27 (7):1570-6.

Supplemental table 3—*Study leadership by method of delivery*

|  | **Telephone*** | **Office** | **Web*** | **Education** |
| --- | --- | --- | --- | --- |
| Total, all studies (n=52) | 18 | 34 | 7 | 14 |
| Physician-led (n=15) | 2 | 13 | 0 | 2 |
| Non-physician-led (n=37) | 17 | 21 | 7 | 13 |

** Pagers included in telephone group; telemedicine units included in web group.*

*NOTE: There were multiple types of interventions in some studies.*

Supplemental table 4—*Articles intervention and outcome, N=52*

*[+ is statistically significant result at p<.05, 0 is no statistically significant result]*

| Author | Year | Intervention1 | Intervention2 | Intervention3 | Surrogate Outcome | Testing or Visit Rates | Quality of life, psychological/ psychsocial, and patient satisfaction | Self-care, including physical activity, diabetes knowledge, and medication adherence | Health Care Utilization and/or Costs | Other | Other - Significant? |
| --- | --- | --- | --- | --- | --- | --- | --- | --- | --- | --- | --- |
| Amoako | 2008 | Telephone |  |  |  |  | + | + |  |  |  |
| Batik | 2008 | Telephone | Education |  |  |  |  | 0 |  |  |  |
| CA group | 2004 | Office | Telephone |  | + |  |  |  |  |  |  |
| Choe | 2005 | Office | Telephone |  | + | + |  |  |  |  |  |
| Clancy | 2003 | Office |  |  | 0 | + |  |  |  |  |  |
| Doucette | 2009 | Office | Education |  | 0 |  |  | + |  |  |  |
| Elder | 2006 | Office | Education |  | 0 |  |  | 0 |  |  |  |
| Gabbay | 2005 | Office | Telephone |  | + | + | + |  |  |  |  |
| Gary | 2003 | Office | Telephone | Education | + |  |  | + |  |  |  |
| Gary | 2004 | Office | Education | Telephone | 0 |  |  |  | 0 |  |  |
| Glasgow | 2000 | Telephone | Education |  | + |  | + | + |  |  |  |
| Glasgow | 2005 | Office | Telephone |  | 0 | + | + | + |  |  |  |
| Hirsh | 2002 | Office |  |  | + |  |  |  | 0 |  |  |
| Hiss | 2007 | Office |  |  | + |  |  |  |  |  |  |
| Ilag | 2003 | Office |  |  | 0 | + |  |  |  |  |  |
| Izquierdo | 2007 | Web |  |  |  |  |  |  |  | Number of medically urgent events | 0 |
| Kennedy | 2006 | Office |  |  | + |  |  |  |  |  |  |
| King | 2009 | Office | Telephone |  | + |  |  |  |  |  |  |
| Krein | 2004 | Telephone | Office |  | 0 |  | + |  | + |  |  |
| Leu | 2005 | Telephone (pager) |  |  | + |  | + |  |  |  |  |
| Lorig | 2008 | Education | Telephone |  | + |  |  | + | 0 |  |  |
| McMahon | 2005 | Web | Education |  | + |  |  |  |  |  |  |
| Nuovo | 2009 | Office |  |  | + |  |  | + |  |  |  |
| O'Connor - A | 2005 | Office |  |  | 0 | 0 |  |  |  |  |  |
| O'Connor - B | 2005 | Office |  |  | + | + |  |  |  |  |  |
| O'Connor - A | 2009 | Office | Education |  | + | + |  |  |  | Medication initiations | 0 |
| O'Connor - B | 2009 | Office |  |  | + | 0 |  |  |  | Number of risky prescribing events | + |
| Odegard | 2005 | Office | Telephone |  | 0 |  |  | 0 |  |  |  |
| Patric | 2006 | Office | Education |  |  | + | 0 |  |  |  |  |
| Pettitt | 2005 | Office |  |  |  |  |  |  |  | Risk of new-onset retinopathy | + |
| Phillips | 2005 | Office |  |  | + |  |  |  |  |  |  |
| Piatt | 2006 | Office |  |  | + |  | + | + |  |  |  |
| Piette | 2001 | Telephone |  |  | + | + | + | + |  |  |  |
| Pitale | 2005 | Office |  |  |  |  | 0 |  |  |  |  |
| Pollard | 2009 | Office |  |  | + | + |  |  |  |  |  |
| Quinn | 2009 | Telephone | Web |  | 0 | 0 | 0 | 0 | 0 |  |  |
| Raji | 2002 | Education |  |  | 0 |  |  | 0 |  |  |  |
| Ralston | 2009 | Web |  |  | + |  |  |  | 0 |  |  |
| Rothman | 2005 | Office | Telephone |  | + |  | 0 | 0 | 0 |  |  |
| Samuel-Hodge | 2009 | Education |  |  | + |  |  |  |  |  |  |
| Scott | 2006 | Office |  |  | + |  | + |  |  |  |  |
| Schmittdiel | 2009 | Office |  |  | + |  |  | 0 |  | Adherence to quality measures and provider attitudes | 0 |
| Sequist | 2005 | Office |  |  |  |  |  |  |  | Adherence to quality measures | 0 |
| Shea | 2006 | Web |  |  | + |  |  |  |  |  |  |
| Shea | 2009 | Web |  |  | + |  |  |  |  |  |  |
| Smith | 2008 | Office |  |  | 0 |  |  |  | + | Provider scores | 0 |
| Stroebel | 2002 | Office |  |  | + | + |  |  |  |  |  |
| Thomas | 2006 | Education |  |  | + |  | 0 | 0 |  |  |  |
| Whitlock | 2000 | Telemedicine unit |  |  | + |  |  |  |  |  |  |
| Walker | 2011 | Education | Telephone |  | + |  |  | 0 |  | Medication possession ratio (adherence) | + |
| Williams | 2007 | Office |  |  | 0 |  | + |  |  |  |  |
| Wolf | 2004 | Office | Education | Telephone | + |  | + |  |  |  |  |

**Supplemental table 5. Subgroup analyses***

|  | **No studies** | **weighted difference in means** | **LL** | **UL** | **p value** |
| --- | --- | --- | --- | --- | --- |
| **A1c** | | | | | |
| **Intervention type** | | | | | |
| education based | 1 | -0.36 | -0.83 | 0.11 | 0.73 |
| Office based | 10 | -0.29 | -0.59 | 0.01 |
| telephone based | 3 | -0.07 | -0.41 | 0.26 |
| Web based | 3 | -0.20 | -0.34 | -0.06 |
| **Length of follow up** | | | | | |
| ≤ 1 year | 5 | -0.19 | -0.27 | -0.12 | 0.50 |
| > 1 year | 12 | -0.30 | -0.58 | -0.01 |
| **Physician led** | | | | | |
| No | 13 | -0.16 | -0.27 | -0.05 | 0.29 |
| Yes | 4 | -0.45 | -0.96 | 0.07 |
| **LDL** | | | | | |
| **Intervention type** | | | | | |
| Office based | 6 | -0.86 | -3.85 | 2.12 | 0.33 |
| Web based | 3 | -4.08 | -9.87 | 1.71 |
| **Length of follow up** | | | | | |
| < 1 year | 1 | -2.70 | -8.63 | 3.23 | 0.91 |
| > 1 year | 8 | -2.30 | -5.78 | 1.18 |
| **Physician led** | | | | | |
| No | 7 | -2.29 | -6.15 | 1.57 | 0.93 |
| Yes | 2 | -2.58 | -7.95 | 2.80 |

* Only feasible analyses are shown. LL and UL and lower and upper limits of 95% confidence intervals

Supplemental figure 1—*Intervention delivery methods and statistically significant outcomes*

Supplemental Figure 2: Study outcomes by leader type

**Search strategy**

EMBASE, Current Contents, Ovid MEDLINE(R), CDSR, CCTR

| **#** | **Searches** | **Results** |
| --- | --- | --- |
| 1 | care management.mp. | 4466 |
| 2 | exp Disease Management/ | 6746 |
| 3 | disease management$.mp. | 9945 |
| 4 | care management$.mp. | 4473 |
| 5 | exp Case Management/ | 6676 |
| 6 | case management$.mp. | 9914 |
| 7 | care model.mp. | 1321 |
| 8 | exp Patient Care Team/ | 44022 |
| 9 | shared care.mp. | 583 |
| 10 | exp Critical Pathways/ | 3252 |
| 11 | critical pathway$.mp. | 3756 |
| 12 | (critical path or critical paths).mp. [mp=title, original title, abstract, name of substance word, subject heading word, unique identifier] | 299 |
| 13 | (clinical pathway$ or clinical path or clinical paths).mp. [mp=title, original title, abstract, name of substance word, subject heading word, unique identifier] | 1369 |
| 14 | exp Patient Care Planning/ | 43990 |
| 15 | exp Primary Nursing Care/ | 1902 |
| 16 | primary nursing care$.mp. | 1920 |
| 17 | exp Primary Health Care/ | 57717 |
| 18 | primary health care$.mp. | 45752 |
| 19 | primary care$.mp. | 48582 |
| 20 | exp "Continuity of Patient Care"/ | 10598 |
| 21 | primary healthcare$.mp. | 1060 |
| 22 | "continuity of patient care".mp. | 10674 |
| 23 | patient care continuity.mp. | 7 |
| 24 | "continuuum of care".mp. | 0 |
| 25 | care continuum.mp. | 215 |
| 26 | "continuity of care".mp. | 2628 |
| 27 | care continuity.mp. | 111 |
| 28 | exp Comprehensive Health Care/ | 163754 |
| 29 | comprehensive health care$.mp. | 6014 |
| 30 | exp Ambulatory Care/ | 40155 |
| 31 | ambulatory care$.mp. | 43479 |
| 32 | outpatient care$.mp. | 2173 |
| 33 | outpatient health service$.mp. | 48 |
| 34 | (clinic visit or clinic visits).mp. [mp=title, original title, abstract, name of substance word, subject heading word, unique identifier] | 3581 |
| 35 | exp Pharmaceutical Services/ | 39051 |
| 36 | pharmaceutical service$.mp. | 6665 |
| 37 | pharmaceutical care$.mp. | 939 |
| 38 | pharmacy service$.mp. | 10906 |
| 39 | pharmacy care$.mp. | 46 |
| 40 | exp Pharmacists/ | 7861 |
| 41 | pharmacist$.mp. | 16801 |
| 42 | (coordinat$ adj2 care).mp. [mp=title, original title, abstract, name of substance word, subject heading word, unique identifier] | 2114 |
| 43 | "process of care".mp. | 1072 |
| 44 | (nurs$ adj2 manag$).mp. [mp=title, original title, abstract, name of substance word, subject heading word, unique identifier] | 6416 |
| 45 | (care team or care teams).mp. [mp=title, original title, abstract, name of substance word, subject heading word, unique identifier] | 45244 |
| 46 | care planning.mp. | 31251 |
| 47 | 1 or 2 or 3 or 4 or 5 or 6 or 7 or 8 or 9 or 10 or 11 or 12 or 13 or 14 or 15 or 16 or 17 or 18 or 19 or 20 or 21 or 22 or 23 or 24 or 25 or 26 or 27 or 28 or 29 or 30 or 31 or 32 or 33 or 34 or 35 or 36 or 37 or 38 or 39 or 40 or 41 or 42 or 43 or 44 or 45 or 46 | 351396 |
| 48 | 1 or 3 or 4 or 5 or 6 or 7 or 9 or 10 or 11 or 12 or 13 or 14 or 15 or 16 or 17 or 18 or 19 or 21 or 22 or 23 or 24 or 25 or 26 or 27 or 29 or 30 or 31 or 32 or 33 or 34 or 35 or 36 or 37 or 38 or 39 or 40 or 41 or 42 or 43 or 44 or 45 or 46 | 295250 |
| 49 | exp Treatment Outcome/ | 417365 |
| 50 | (outcome$ or effectiveness or efficacy).mp. [mp=title, original title, abstract, name of substance word, subject heading word, unique identifier] | 1258440 |
| 51 | exp Hemoglobin A, Glycosylated/ or a1c.mp. | 17112 |
| 52 | glycosylated hemoglobin$.mp. | 3660 |
| 53 | glycated hemoglobin$.mp. | 1356 |
| 54 | exp Lipids/ | 780677 |
| 55 | lipid$.mp. | 321295 |
| 56 | exp Hyperlipidemias/ | 48665 |
| 57 | hyperlip$.mp. | 39114 |
| 58 | lipemia$.mp. | 1227 |
| 59 | exp Hypoglycemia/ | 18361 |
| 60 | hypoglycemia$.mp. | 25481 |
| 61 | glycem$.mp. | 15479 |
| 62 | exp Patient Satisfaction/ | 42097 |
| 63 | (satisf$ or preference$).mp. | 258050 |
| 64 | exp "Quality of Life"/ | 79221 |
| 65 | economics.hw. | 48651 |
| 66 | economics.sh. | 25705 |
| 67 | (economic$ or cost or costs).mp. [mp=title, original title, abstract, name of substance word, subject heading word, unique identifier] | 384970 |
| 68 | mortality.mp. | 340267 |
| 69 | blood pressure.mp. or exp Blood Pressure/ | 312190 |
| 70 | (quality adj2 life).mp. [mp=title, original title, abstract, name of substance word, subject heading word, unique identifier] | 120197 |
| 71 | ((pulse or systolic or diastolic) adj pressure).mp. [mp=title, original title, abstract, name of substance word, subject heading word, unique identifier] | 21920 |
| 72 | 49 or 50 or 51 or 52 or 53 or 54 or 55 or 56 or 57 or 58 or 59 or 60 or 61 or 62 or 63 or 64 or 65 or 66 or 67 or 68 or 69 or 70 or 71 | 3149638 |
| 73 | exp *Diabetes Mellitus/rh, dt, ec, th, rt, mo, pc, su, dh [Rehabilitation, Drug Therapy, Economics, Therapy, Radiotherapy, Mortality, Prevention & Control, Surgery, Diet Therapy] | 55098 |
| 74 | (diabetes or iddm or niddm).ti. | 110534 |
| 75 | [exp *diabetes mellitus/dt, rh, dm, su, rt, pc, th [Drug Therapy, Rehabilitation, Disease Management, Surgery, Radiotherapy, Prevention, Therapy]] | 0 |
| 76 | 73 or 74 or 75 | 137843 |
| 77 | exp controlled study/ | 0 |
| 78 | exp evidence based medicine/ | 35730 |
| 79 | evidence-based.mp. | 51354 |
| 80 | ((control$ or randomized) adj2 (study or studies or trial or trials)).mp. [mp=title, original title, abstract, name of substance word, subject heading word, unique identifier] | 619809 |
| 81 | meta analysis/ | 23124 |
| 82 | meta-analys$.mp. | 39896 |
| 83 | exp "systematic review"/ | 0 |
| 84 | systematic review$.mp. | 19357 |
| 85 | exp Guideline/ or exp Practice Guideline/ | 19554 |
| 86 | guideline$.ti. | 33800 |
| 87 | 77 or 78 or 79 or 80 or 81 or 82 or 83 or 84 or 85 or 86 | 728368 |
| 88 | [from 47 keep 1-1765497] | 0 |
| 89 | 47 not 88 | 351396 |
| 90 | 89 or 48 | 351396 |
| 91 | 72 and 76 and 87 and 90 | 819 |
| 92 | [from 47 keep 1824406-2181235] | 0 |
| 93 | 72 and 76 and 87 and 92 | 0 |
| 94 | limit 93 to (controlled clinical trial or guideline or meta analysis or randomized controlled trial) | 0 |
| 95 | 91 or 94 | 819 |
| 96 | limit 95 to ("all adult (19 plus years)" or "young adult (19 to 24 years)" or "adult (19 to 44 years)" or "young adult and adult (19-24 and 19-44)" or "middle age (45 to 64 years)" or "middle aged (45 plus years)" or "all aged (65 and over)" or "aged (80 and over)") | 543 |
| 97 | limit 96 to (adult <18 to 64 years> or aged <65+ years>) [Limit not valid in Ovid MEDLINE(R); records were retained] | 543 |
| 98 | limit 97 to yr="2000 -Current" | 453 |
| 99 | remove duplicates from 98 | 423 |
| 100 | limit 99 to (editorial or letter or news) | 0 |
| 101 | limit 100 to (editorial or letter or news) | 0 |
| 102 | 99 not 100 not 101 | 423 |
| 103 | [from 102 keep 1-777] | 0 |
| 104 | [from 103 keep 1-777] | 0 |
| 105 | 102 not 103 | 423 |
| 106 | from 105 keep 1-217 | 217 |
| 107 | from 106 keep 1-217 | 217 |
| 108 | 105 not 106 | 206 |
| 109 | [from 108 keep 1-423] | 0 |
| 110 | [from 109 keep 1-423] | 0 |
| 111 | 108 not 109 | 206 |
| 112 | from 111 keep 1-23 | 23 |
| 113 | from 112 keep 1 | 1 |

CINAHL

| Search ID# | Search Terms | Search Options | Last Run Via | Results |
| --- | --- | --- | --- | --- |
| S84 | S41 and S66 and S71 and S82 | Limiters - Published Date from: 20000101-20091231; Exclude MEDLINE records; Age Groups: All Adult  Search modes - Boolean/Phrase | Interface - EBSCOhost  Search Screen - Advanced Search  Database - CINAHL | 21 |
| S83 | S41 and S66 and S71 and S82 | Search modes - Boolean/Phrase | Interface - EBSCOhost  Search Screen - Advanced Search  Database - CINAHL | Display |
| S82 | S72 or S73 or S74 or S75 or S76 or S77 or S78 or S79 or S80 or S81 | Search modes - Boolean/Phrase | Interface - EBSCOhost  Search Screen - Advanced Search  Database - CINAHL | Display |
| S81 | TI Guideline* | Search modes - Boolean/Phrase | Interface - EBSCOhost  Search Screen - Advanced Search  Database - CINAHL | Display |
| S80 | (MH "Practice Guidelines") | Search modes - Boolean/Phrase | Interface - EBSCOhost  Search Screen - Advanced Search  Database - CINAHL | Display |
| S79 | systematic review* | Search modes - Boolean/Phrase | Interface - EBSCOhost  Search Screen - Advanced Search  Database - CINAHL | Display |
| S78 | (MH "Systematic Review") | Search modes - Boolean/Phrase | Interface - EBSCOhost  Search Screen - Advanced Search  Database - CINAHL | Display |
| S77 | meta analys* | Search modes - Boolean/Phrase | Interface - EBSCOhost  Search Screen - Advanced Search  Database - CINAHL | Display |
| S76 | (MH "Meta Analysis") | Search modes - Boolean/Phrase | Interface - EBSCOhost  Search Screen - Advanced Search  Database - CINAHL | Display |
| S75 | "randomized study" or "randomized studies" or "randomized trial" or "randomized trials" | Search modes - Boolean/Phrase | Interface - EBSCOhost  Search Screen - Advanced Search  Database - CINAHL | Display |
| S74 | "controlled study" or "controlled studies" or "controlled trial" or "controlled trials" | Search modes - Boolean/Phrase | Interface - EBSCOhost  Search Screen - Advanced Search  Database - CINAHL | Display |
| S73 | evidence based | Search modes - Boolean/Phrase | Interface - EBSCOhost  Search Screen - Advanced Search  Database - CINAHL | Display |
| S72 | (MH "Medical Practice, Evidence-Based") | Search modes - Boolean/Phrase | Interface - EBSCOhost  Search Screen - Advanced Search  Database - CINAHL | Display |
| S71 | S67 or S68 or S69 or S70 | Search modes - Boolean/Phrase | Interface - EBSCOhost  Search Screen - Advanced Search  Database - CINAHL | Display |
| S70 | TI niddm | Search modes - Boolean/Phrase | Interface - EBSCOhost  Search Screen - Advanced Search  Database - CINAHL | Display |
| S69 | TI iddm | Search modes - Boolean/Phrase | Interface - EBSCOhost  Search Screen - Advanced Search  Database - CINAHL | Display |
| S68 | TI Diabetes | Search modes - Boolean/Phrase | Interface - EBSCOhost  Search Screen - Advanced Search  Database - CINAHL | Display |
| S67 | (MM "Diabetes Mellitus+") | Search modes - Boolean/Phrase | Interface - EBSCOhost  Search Screen - Advanced Search  Database - CINAHL | Display |
| S66 | (S42 or S43 or S44 or S45 or S46 or S47 or S48 or S49 or S50 or S51 or S52 or S53 or S54 or S55 or S56 or S57 or S58 or S59 or S60 or S61 or S62 or S63 or S64 or S65) | Search modes - Boolean/Phrase | Interface - EBSCOhost  Search Screen - Advanced Search  Database - CINAHL | Display |
| S65 | diastolic pressure | Search modes - Boolean/Phrase | Interface - EBSCOhost  Search Screen - Advanced Search  Database - CINAHL | Display |
| S64 | systolic pressure | Search modes - Boolean/Phrase | Interface - EBSCOhost  Search Screen - Advanced Search  Database - CINAHL | Display |
| S63 | pulse pressure | Search modes - Boolean/Phrase | Interface - EBSCOhost  Search Screen - Advanced Search  Database - CINAHL | Display |
| S62 | ("Blood Pressure") or (MH "Blood Pressure+") | Search modes - Boolean/Phrase | Interface - EBSCOhost  Search Screen - Advanced Search  Database - CINAHL | Display |
| S61 | ("mortality") or (MH "Mortality+") | Search modes - Boolean/Phrase | Interface - EBSCOhost  Search Screen - Advanced Search  Database - CINAHL | Display |
| S60 | economic* or cost or costs | Search modes - Boolean/Phrase | Interface - EBSCOhost  Search Screen - Advanced Search  Database - CINAHL | Display |
| S59 | (MH "Quality of Life+") | Search modes - Boolean/Phrase | Interface - EBSCOhost  Search Screen - Advanced Search  Database - CINAHL | Display |
| S58 | Quality of Life | Search modes - Boolean/Phrase | Interface - EBSCOhost  Search Screen - Advanced Search  Database - CINAHL | Display |
| S57 | satisf* or preference* | Search modes - Boolean/Phrase | Interface - EBSCOhost  Search Screen - Advanced Search  Database - CINAHL | Display |
| S56 | (MH "Patient Satisfaction") | Search modes - Boolean/Phrase | Interface - EBSCOhost  Search Screen - Advanced Search  Database - CINAHL | Display |
| S55 | glycem* | Search modes - Boolean/Phrase | Interface - EBSCOhost  Search Screen - Advanced Search  Database - CINAHL | Display |
| S54 | Hypoglycemia* | Search modes - Boolean/Phrase | Interface - EBSCOhost  Search Screen - Advanced Search  Database - CINAHL | Display |
| S53 | (MH "Hypoglycemia+") | Search modes - Boolean/Phrase | Interface - EBSCOhost  Search Screen - Advanced Search  Database - CINAHL | Display |
| S52 | lipemia* | Search modes - Boolean/Phrase | Interface - EBSCOhost  Search Screen - Advanced Search  Database - CINAHL | Display |
| S51 | hyperlip* | Search modes - Boolean/Phrase | Interface - EBSCOhost  Search Screen - Advanced Search  Database - CINAHL | Display |
| S50 | (MH "Hyperlipidemia+") | Search modes - Boolean/Phrase | Interface - EBSCOhost  Search Screen - Advanced Search  Database - CINAHL | Display |
| S49 | Lipid* | Search modes - Boolean/Phrase | Interface - EBSCOhost  Search Screen - Advanced Search  Database - CINAHL | Display |
| S48 | (MH "Lipids+") | Search modes - Boolean/Phrase | Interface - EBSCOhost  Search Screen - Advanced Search  Database - CINAHL | Display |
| S47 | glycated hemoglobin* | Search modes - Boolean/Phrase | Interface - EBSCOhost  Search Screen - Advanced Search  Database - CINAHL | Display |
| S46 | a1c | Search modes - Boolean/Phrase | Interface - EBSCOhost  Search Screen - Advanced Search  Database - CINAHL | Display |
| S45 | glycosylated hemoglobin* | Search modes - Boolean/Phrase | Interface - EBSCOhost  Search Screen - Advanced Search  Database - CINAHL | Display |
| S44 | (MH "Hemoglobin A, Glycosylated") | Search modes - Boolean/Phrase | Interface - EBSCOhost  Search Screen - Advanced Search  Database - CINAHL | Display |
| S43 | outcome* or effectiveness or efficacy | Search modes - Boolean/Phrase | Interface - EBSCOhost  Search Screen - Advanced Search  Database - CINAHL | Display |
| S42 | (MH "Treatment Outcomes+") | Search modes - Boolean/Phrase | Interface - EBSCOhost  Search Screen - Advanced Search  Database - CINAHL | Display |
| S41 | S1 or S2 or S3 or S4 or S5 or S6 or S7 or S8 or S9 or S10 or S11 or S12 or S13 or S14 or S15 or S16 or S17 or S18 or S19 or S20 or S21 or S22 or S23 or S24 or S25 or S26 or S27 or S28 or S29 or S30 or S31 or S32 or S33 or S34 or S35 or S36 or S37 or S38 or S39 or S40 | Search modes - Boolean/Phrase | Interface - EBSCOhost  Search Screen - Advanced Search  Database - CINAHL | Display |
| S40 | care planning | Search modes - Boolean/Phrase | Interface - EBSCOhost  Search Screen - Advanced Search  Database - CINAHL | Display |
| S39 | care team or care teams | Search modes - Boolean/Phrase | Interface - EBSCOhost  Search Screen - Advanced Search  Database - CINAHL | Display |
| S38 | "care team or care teams" | Search modes - Boolean/Phrase | Interface - EBSCOhost  Search Screen - Advanced Search  Database - CINAHL | Display |
| S37 | nurs* N2 manag* | Search modes - Boolean/Phrase | Interface - EBSCOhost  Search Screen - Advanced Search  Database - CINAHL | Display |
| S36 | process of care | Search modes - Boolean/Phrase | Interface - EBSCOhost  Search Screen - Advanced Search  Database - CINAHL | Display |
| S35 | coordinat* N2 care | Search modes - Boolean/Phrase | Interface - EBSCOhost  Search Screen - Advanced Search  Database - CINAHL | Display |
| S34 | (MH "Pharmacists") | Search modes - Boolean/Phrase | Interface - EBSCOhost  Search Screen - Advanced Search  Database - CINAHL | Display |
| S33 | pharmacy care* | Search modes - Boolean/Phrase | Interface - EBSCOhost  Search Screen - Advanced Search  Database - CINAHL | Display |
| S32 | pharmacy service* | Search modes - Boolean/Phrase | Interface - EBSCOhost  Search Screen - Advanced Search  Database - CINAHL | Display |
| S31 | pharmaceutical care* | Search modes - Boolean/Phrase | Interface - EBSCOhost  Search Screen - Advanced Search  Database - CINAHL | Display |
| S30 | Pharmaceutical Service* | Search modes - Boolean/Phrase | Interface - EBSCOhost  Search Screen - Advanced Search  Database - CINAHL | Display |
| S29 | clinic visit or clinic visits | Search modes - Boolean/Phrase | Interface - EBSCOhost  Search Screen - Advanced Search  Database - CINAHL | Display |
| S28 | outpatient health service* | Search modes - Boolean/Phrase | Interface - EBSCOhost  Search Screen - Advanced Search  Database - CINAHL | Display |
| S27 | outpatient care* | Search modes - Boolean/Phrase | Interface - EBSCOhost  Search Screen - Advanced Search  Database - CINAHL | Display |
| S26 | Ambulatory Care* | Search modes - Boolean/Phrase | Interface - EBSCOhost  Search Screen - Advanced Search  Database - CINAHL | Display |
| S25 | (MH "Ambulatory Care") | Search modes - Boolean/Phrase | Interface - EBSCOhost  Search Screen - Advanced Search  Database - CINAHL | Display |
| S24 | Comprehensive Health Care* | Search modes - Boolean/Phrase | Interface - EBSCOhost  Search Screen - Advanced Search  Database - CINAHL | Display |
| S23 | care continuity | Search modes - Boolean/Phrase | Interface - EBSCOhost  Search Screen - Advanced Search  Database - CINAHL | Display |
| S22 | continuity of care | Search modes - Boolean/Phrase | Interface - EBSCOhost  Search Screen - Advanced Search  Database - CINAHL | Display |
| S21 | "continuum of care" | Search modes - Boolean/Phrase | Interface - EBSCOhost  Search Screen - Advanced Search  Database - CINAHL | Display |
| S20 | patient care continuity | Search modes - Boolean/Phrase | Interface - EBSCOhost  Search Screen - Advanced Search  Database - CINAHL | Display |
| S19 | primary healthcare* | Search modes - Boolean/Phrase | Interface - EBSCOhost  Search Screen - Advanced Search  Database - CINAHL | Display |
| S18 | ("Continuity of Patient Care") or (MH "Continuity of Patient Care+") | Search modes - Boolean/Phrase | Interface - EBSCOhost  Search Screen - Advanced Search  Database - CINAHL | Display |
| S17 | primary health care* or primary care* | Search modes - Boolean/Phrase | Interface - EBSCOhost  Search Screen - Advanced Search  Database - CINAHL | Display |
| S16 | primary nursing care* | Search modes - Boolean/Phrase | Interface - EBSCOhost  Search Screen - Advanced Search  Database - CINAHL | Display |
| S15 | (MH "Primary Health Care") or (MH "Primary Nursing") | Search modes - Boolean/Phrase | Interface - EBSCOhost  Search Screen - Advanced Search  Database - CINAHL | Display |
| S14 | (MH "Patient Care Plans+") or (MH "Nursing Care Plans+") | Search modes - Boolean/Phrase | Interface - EBSCOhost  Search Screen - Advanced Search  Database - CINAHL | Display |
| S13 | clinical pathway* or clinical path or clinical paths | Search modes - Boolean/Phrase | Interface - EBSCOhost  Search Screen - Advanced Search  Database - CINAHL | Display |
| S12 | Critical Paths | Search modes - Boolean/Phrase | Interface - EBSCOhost  Search Screen - Advanced Search  Database - CINAHL | Display |
| S11 | Critical Path | Search modes - Boolean/Phrase | Interface - EBSCOhost  Search Screen - Advanced Search  Database - CINAHL | Display |
| S10 | Critical Pathway* | Search modes - Boolean/Phrase | Interface - EBSCOhost  Search Screen - Advanced Search  Database - CINAHL | Display |
| S9 | (MH "Critical Path") | Search modes - Boolean/Phrase | Interface - EBSCOhost  Search Screen - Advanced Search  Database - CINAHL | Display |
| S8 | "shared care" | Search modes - Boolean/Phrase | Interface - EBSCOhost  Search Screen - Advanced Search  Database - CINAHL | Display |
| S7 | (MH "Multidisciplinary Care Team+") | Search modes - Boolean/Phrase | Interface - EBSCOhost  Search Screen - Advanced Search  Database - CINAHL | Display |
| S6 | "care model" | Search modes - Boolean/Phrase | Interface - EBSCOhost  Search Screen - Advanced Search  Database - CINAHL | Display |
| S5 | Case Management* | Search modes - Boolean/Phrase | Interface - EBSCOhost  Search Screen - Advanced Search  Database - CINAHL | Display |
| S4 | (MH "Case Management") | Search modes - Boolean/Phrase | Interface - EBSCOhost  Search Screen - Advanced Search  Database - CINAHL | Display |
| S3 | care Management* | Search modes - Boolean/Phrase | Interface - EBSCOhost  Search Screen - Advanced Search  Database - CINAHL | Display |
| S2 | "Disease Management" | Search modes - Boolean/Phrase | Interface - EBSCOhost  Search Screen - Advanced Search  Database - CINAHL | Display |
| S1 | (MH "Disease Management") | Search modes - Boolean/Phrase | Interface - EBSCOhost  Search Screen - Advanced Search  Database - CINAHL | Display |
